# Thinking hierarchically about posttraumatic response: Commentary on Lehinger et al. (2022)

**DOI:** 10.1002/jts.22891

**Published:** 2022-10-31

**Authors:** Matthew M. Yalch, Alana R. Gallagher, Kayleigh N. Watters

**Affiliations:** ^1^ Department of Psychology Palo Alto University Palo Alto California USA; ^2^ Department of Psychiatry and Behavioral Sciences Stanford University School of Medicine Stanford California USA

## Abstract

Lehinger et al.’s (2022) study on the associations between posttraumatic stress symptoms, posttraumatic cognitions, and alcohol use in sexual assault survivors extends previous research on posttraumatic response to sexual trauma. The study is useful for these purposes but it also raises other interesting questions about the nature of posttraumatic response and the structure of psychopathology more generally. In this commentary, we describe Lehinger et al.’s (2022) study and its findings and discuss their potential relevance for emerging transdiagnostic, hierarchical models of psychopathology.

Lehinger et al.’s (2022) study on the direct and indirect associations between posttraumatic stress symptoms (PTSS), posttraumatic cognitions, and alcohol use among sexual assault survivors is useful in many ways. It confirms previous work showing a link between PTSS and posttraumatic cognitions of sexual trauma (e.g., Kline et al., 2021). It also extends research (e.g., Jayawickreme et al., 2012) on the associations between PTSS, posttraumatic cognitions, and alcohol use, parsing drinking from its consequences. Relatedly, the study's findings highlight the role of self‐blame in women whose sexual assaults involved alcohol, which has clear implications for interventions with sexual assault survivors.

The primary findings of the study are important in and of themselves. However, by examining some of the less‐emphasized results, there may be even more to learn. For example, considering the results at the bivariate level, there is a strong association, *r*_mean_ = .43, between PTSS, posttraumatic cognitions, and drinking consequences. This suggests that these variables have a high degree of overlap in problematic posttraumatic response following sexual assault. It may, thus, be worthwhile to conceptualize posttraumatic response differently. One way to do this is to consider a broader range of constructs related to posttraumatic response. For instance, survivors of sexual assault and other traumatic stressors experience forms of posttraumatic distress other than PTSS (e.g., depression, anxiety, and dissociation, which often co‐occur with PTSS).

These associations between different kinds of posttraumatic distress and posttraumatic cognitions may also reflect one or more underlying dimensions of broader psychopathology. This general idea is the driving force behind a movement in the broader psychopathology literature examining the hierarchical taxonomy of psychopathology (HiTOP; Kotov et al., 2021). HiTOP conceptualizes psychopathology not as discrete disorders but rather as spectra of psychopathology arranged hierarchically: An overall distress factor at the top, followed by an echelon of broader spectra of more specific domains of problems including internalizing and externalizing pathology, followed by more specific domains of problems, and so on.

There is evidence of shared dimensions underlying posttraumatic distress and posttraumatic cognitions not only in strong bivariate associations but also in the associations between these different aspects of posttraumatic distress and personality traits, which recent research suggests provides the structural foundation for psychopathology. For example, there is robust research on the association between personality traits and both PTSS, most notably neuroticism, and problems related to substance use, most notably disinhibition, as well as a host of other forms of psychopathology, posttraumatic and otherwise (Kotov et al., 2010). The results of several recent studies also suggest comparable associations between personality and posttraumatic cognitions regarding sexual assault (Yalch et al., 2022; see also Gallagher et al., 2022). Specifically, research indicates that neuroticism is associated with a broad range of posttraumatic cognitions, particularly self‐blame, whereas antagonism and psychoticism are hallmarks of negative cognitions about the world. Based on these associations, PTSS, posttraumatic cognitions, and alcohol‐related problems might be considered not as separate constructs but as forms of posttraumatic response that represent different levels of specificity (see Figure 1).

**FIGURE 1:**
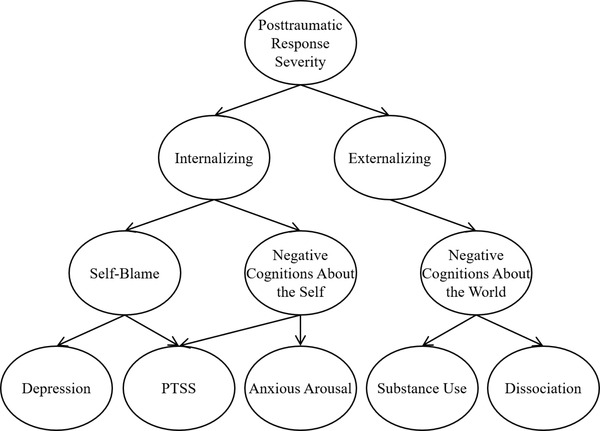
A potential hierarchical structure of posttraumatic response *Note*: PTSS = posttraumatic stress symptoms.

There are some benefits to conceptualizing posttraumatic response hierarchically. First, thinking about these different aspects of posttraumatic response as structurally related attenuates problems of causality inherent in cross‐sectional research designs, which Lehinger et al. (2022) acknowledged and is a common limitation of many studies. Taking a hierarchical approach is also useful for clinical assessment and intervention, and there is emerging literature on how to use this approach to identify a patient's most impairing domain of problems and intervene at a level of specificity that matches their idiosyncratic presentation (Hopwood et al., 2020). In the case of posttraumatic distress, the domain of problems may be narrow (e.g., depression associated with self‐blame following an isolated sexual assault) or broad (e.g., high levels of depression, anxiety, and suicidality; prominent identity disturbance; and polysubstance use following lifelong interpersonal trauma).

Taking such an approach would not be new for trauma‐focused clinicians (see Gutner et al., 2016). The heterogeneity in both the type and severity of posttraumatic response, especially in cases of complex trauma, has long been recognized, and taking a transdiagnostic approach to treating trauma survivors is increasingly gaining traction (e.g., O'Donnell et al., 2021; Varkovitzky et al., 2018). However, current transdiagnostic conceptualizations of trauma and its treatment have not typically taken a hierarchical approach. Adopting such a perspective would be more consistent with emerging research on the structure of psychopathology and could be beneficial for trauma survivors by providing a more individualized approach to assessment, case conceptualization, and treatment.
